# Discordance between lifestyle-related health behaviors and beliefs of urban mainland Chinese: A questionnaire study with implications for targeting health education

**DOI:** 10.3934/publichealth.2019.1.49

**Published:** 2019-02-20

**Authors:** Peng Wang, Zhenyi Li, Alice Jones, Michael E. Bodner, Elizabeth Dean

**Affiliations:** 1 School of Foreign Studies, Central University of Finance and Economics, Beijing, China, and formerly visiting scholar, School of Communication and Culture, Royal Roads University, Victoria, British Columbia, Canada; 2 School of Communication and Culture, Royal Roads University, Victoria, British Columbia, Canada; 3 Alice Jones, Discipline of Physiotherapy, Faculty of Health Sciences, The University of Sydney, Sydney, Australia; 4 School of Human Kinetics, Trinity Western University, Langley, British Columbia, Canada; 5 Department of Physical Therapy, Faculty of Medicine, University of British Columbia, Vancouver, Canada

**Keywords:** health promotion education, lifestyle-related behaviors, lifestyle-related beliefs, mainland Chinese, non-communicable diseases

## Abstract

**Background:**

Morbidity and mortality in China are increasingly associated with lifestyle behaviors, e.g., smoking, poor nutritional choices, and physical inactivity. Lifestyle-related non-communicable diseases (e.g., hypertension, stroke, heart disease, lung disease) are at critical levels globally, in turn their socioeconomic burdens. Knowledge of lifestyle-related health behaviors and beliefs of mainland Chinese would help inform the design and targeting of cost-effective health education for individuals and campaigns in the interests of promoting and protecting health, and preventing disease. This study's objective was to describe the lifestyle behaviors and beliefs of a sample of urban mainland Chinese, and their congruence with evidence-based guidelines for maximal health.

**Methods:**

A cross-sectional interview questionnaire study was conducted in which 835 mainland Chinese (55% men, 45% women) from four urban areas participated.

**Results:**

About half (52%) reported smoking to some degree with 21% being habitual smokers; 33% being above average weight; 62.1% met physical activity guidelines for health benefits; 92% being sedentary for 5.8 ± 3.40 h/d; 66% experiencing moderate/high stress; and sleeping 7.1 ± 1.31 h nightly with 35% reporting sleeping poorly. When standard serving sizes were considered, daily consumption of grains, fruits, and vegetables was reported to be consistent with dietary recommendations for good health, however, added salt (3.7 ± 7.42 tsp) and sugar (3.9 ± 12.99 tsp) exceeded recommendations. Life stress was rated moderate by 59.6% of respondents, with personal and family health stresses ranking highest (43% and 55%, respectively). Regarding beliefs about importance of lifestyle behaviors to health, respondents' understanding was not consistent with evidence-based recommendations. Only 64% of participants believed smoking abstinence is highly important to health; 56% regular exercise; and 37% consumption of whole grains, 62% fruit and vegetables; and 54% maintaining a healthy body weight.

**Conclusion:**

To be congruent with established guidelines for healthy living, health promotion and disease prevention education for individuals and public campaigns warrants targeting health knowledge and beliefs of urban Chinese as well as lifestyle-related health behaviors. The roles of gender, education and living rurally, on lifestyle behaviors and beliefs of the Chinese, warrant elucidation.

## 1. Introduction

Over recent decades, lifestyle-related non-communicable diseases (NCDs) have become increasingly prevalent with economic development in China [1],[2]. This has been compounded by the aging of the Chinese population [3]. Eighty-three per cent of all deaths in China are now attributed to NCDs with 38% being secondary to vascular diseases [2]. Given that the Chinese population exceeds 1.3 billion, even a small proportion of people affected by NCDs translates into considerable burden in terms of direct and indirect health care and associated costs, and burden to the family and health care system.

Prevention and control of lifestyle-related modifiable risk factors, such as healthful changes related to smoking, diet, body weight, and exercise can reduce the incidence of vascular and metabolic disease and other disabling conditions as well as may reverse these [4]–[6]. Since the mid-1990s, upward trends in metabolic risk factors in the Chinese population have been reported by the World Health Organization, specifically, systolic blood pressure, fasting blood sugar, body mass index (BMI), and total cholesterol [1]. The Chinese government has taken positive steps toward addressing this crisis, however authorities are strongly urging the government to develop the essential evidence-based disease control policies and funding priorities [7].

Recently, we have reported on the low level of knowledge of over 800 mainland Chinese about the warning signs of stroke, emergency response if someone appears to be having a stroke, and where they would seek out information about stroke [8]. Health knowledge however is but one component when attempting to effect lifestyle behavior change. The Health Belief Model advocates that understanding a person's lifestyle-related behaviors should precede the actual goal of changing these [9]. Therefore, an understanding of the lifestyle-related health behaviors as well as beliefs of mainland Chinese would help inform the design and targeting of education campaigns for preventing NCDs such as stroke. Similar studies have been reported on lifestyle practices and beliefs associated with NCDs, across cultures including Singapore [10], Kuwait [11],[12], and Saudi Arabia [13], and various ethnic groups at risk in Canada including those of Chinese origin and ethnicity [14],[15].

The purpose of the present study was to examine the concordance between lifestyle-related health behaviors and beliefs, i.e., regarding smoking, diet, activity and inactivity, and stress and sleep, in urban mainland Chinese, and the concordance of their behaviors and beliefs with established recommendations for health. Such knowledge may help to design, target, and implement health promotion and disease prevention education programs for individuals and the general public in China, with a view to protect health, and reduce the prevalence of lifestyle-related NCDs and their risk factors, and their associated social and economic burdens.

## 2. Methods

### 2.1. Study design and participants

A cross-sectional descriptive study was designed based on a structured face-to-face interview questionnaire. The study was reviewed and approved through the ethics review process of the participating institutions. The participants provided signed informed consent prior to their data being included. The target population included adult residents in four urban areas in the Chinese provinces of Hubei, Liaoning, Hebei, and Shanxi. The valid sample size was 835. Of the total of 846, 11 respondents failed to give their ages or volunteers were under 18 years of age. Interviewers (n = 80) were qualified health professionals (e.g., physicians, nurses, and social workers). At the time of this study, these individuals were students in masters of physical therapy programs. As part of their professional training related to practice competencies, these students were trained to interview people about their lifestyle-related health behaviors and beliefs. Standardized interviewing technique as a clinical competency helped ensure a standardized procedure was instituted including how to address questions. We selected a purposive snowballing sampling method in that student interviewers were asked to recruit at least 10 individuals for interviewing (range 10 to 15). These individuals could be on or off campus, with an emphasis on those they did not know personally. Student interviewers were instructed to inform all respondents not only about the nature of the questionnaire, but also that no identifying information was required. Interviews were conducted within two weeks. Each interview was 30 minutes in duration.

### 2.2. Questionnaire

A structured questionnaire was developed based on previous work conducted in Canada [14],[15] Kuwait [11],[12], Saudi Arabia [13] and Singapore [10]. We conducted the questionnaire by interview to maximize the quality and completeness of the data collection. The interviewers were not on the research team which helped minimize potential conflict of interest. The questionnaire consisted of four sections with four open-ended questions in section one, and 48 closed-ended questions in sections two, three and four. Section one asked questions about common risk factors for stroke, warning signs, knowledge of emergency response, and where to seek resources. The data from these questions have been published as a qualitative report [8]. The present study focuses on participants' responses to the closed-ended questions in the remaining three sections. Sections two and three included questions about lifestyle-related health behaviors and beliefs, respectively, regarding smoking, diet, physical activity and inactivity, stress, and sleep (Appendix). We based the questionnaire used in the Chinese context in this study on our previous work [10]–[15] which reflected the literature with respect to questions about smoking behavior, food categories and serving sizes and quantities, and physical activity and exercise definitions as well as a definition for sedentary behavior. This was essential to ensure that the information given to respondents was consistent across interviewers. The final section, section four, included questions about respondents' demographic characteristics. With respect to BMI, we asked respondents' their heights and weights so that BMI could be calculated, but we also asked them to identify their perception of their body mass as healthy, under or overweight.

The questionnaire was pre-tested on a convenience sample (n = 7) and modifications were made to enhance clarity of the items. More unambiguous mutually exclusive choices were added for items with multiple response options. In the interest of cultural relevancy, the unit of consumption in the dietary section was set to grams. Two proficiently bilingual individuals independently translated the questionnaire into Mandarin. The questionnaire was tested for clarity and comprehension on a sample of four bilingual individuals and revised accordingly.

### 2.3. Statistical analysis

All variables were summarized using standard descriptive statistics including means, standard deviations, and frequencies. Normality of distribution of continuous variables was assessed using the Kolmogorov-Smirnov test. Chi-square tests compared the frequency of those categories of interest. Kendall's Tau-b Tests were performed to determine the association between ordered variables. Mann-Whitney U tests assessed gender differences for physical activity variables. The Kruskal-Wallis test for independent samples assessed level of significance for variables with non-normal distributions. Where statistical significance was observed for the Kruskal-Wallis test, the Mann-Whitney U test with Bonferroni corrections was used for post-hoc analysis. All *p* values were two-sided and alpha was set at 0.05. The data were analyzed using Statistical Package for Social Sciences (SPSSx Windows version 22.0; Chicago, IL, USA).

We selected cut-points for recommendations for maximal health status that are established, evidence-based, and commonly reported [12]. These include no amount of smoking is consistent with maximal health [16],[17]. With respect to healthy nutrition [18],[19], recommendations for the Chinese are 300 to 500 g daily of cereals/multi-grains, at least 500 g of fruits and vegetables, no more than 125 to 200 g animal protein; no more than 25 g of fats and oils; and limited, if any, added sugar and salt. For the Asian population the recommended BMI ranges from 18.5 to 24.9 kg m^2^ [20]. With respect to physical activity for health, the international recommendation is 150 minutes/week of moderately intense exercise or 75 minutes of intense exercise [21],[22]. For health, 8–10 hours of sleep a night are recommended [23] and stress levels should remain manageable and relatively low to avoid chronic unabated stress and its health consequences [24].

## 3. Results

### 3.1. Respondents' demographics

Table 1 shows the demographic and personal characteristics of the sample. Briefly, the mean age (±SD) was 45.7 ± 16.63 y. Of the total of 835 respondents (n = 456 men, 55%; n = 371 women, 45%), 274 (33.1%) were between 18 and 34 y, 177 (21.4%) between 35 and 49 y, 232 (28.1%) between 50 and 64 y, and 144 (17.4%) ≥ 65 y. Participants over 35 y of age constituted 66.9% of the sample. A similar distribution of age appeared for both genders. The mean BMI was 23.0 ± 4.27 kg m^2^; 655 people (78.4%) were below a BMI of 25, 155 (18.6%) were between 25 and 30, with 22 (2.6%) over 30. In terms of education, 468 respondents (56.1%) held a college/university degree; 299 (35.8%) technical or trade school diploma; and only 62 (7.4%) had no formal education including elementary school education.

**Table 1.** Demographic characteristics of participants (n = 827 with complete demographic information) (n, %).

	Total (n = 827)		Men (n = 456)		Women (n = 371)	
	n	%	n	%	n	%
Age (y)						
18–34	274	33.1	160	35.1	114	30.7
35–49	177	21.4	92	20.2	85	22.9
50–64	232	28.1	132	28.9	100	27.0
≥65	144	17.4	72	15.8	72	19.4
Body Mass Index (kg/m^2^)						
<25	655	78.4	353	77.9	295	79.5
≥25 to ≤30	155	18.6	91	20.1	64	17.3
>30	22	2.6	9	2	12	3.2
Education						
None or elementary school	62	7.4	29	6.4	33	8.9
Technical or trade school; high school	299	35.8	149	33.0	144	39.0
College/university diploma; postgraduate degree	468	56.1	274	60.0	192	52.0
Type of Work						
Homemaker	80	9.6	17	3.8	58	15.7
Not employed	158	18.9	96	21.3	62	16.8
Retired	228	27.3	123	27.3	104	28.1
Other	26	3.1	12	2.7	13	3.5
Employed	336	40.2	202	44.9	133	35.9
Occupation						
Business, finance and administration	118	14.1	67	15.4	51	15.0
Management	49	5.9	33	7.6	16	4.7
Professional	410	49.1	243	56	161	47.4
Sales and service	89	10.7	36	8.3	52	15.3
Trades, transport and equipment Operator	39	4.7	27	6.2	11	3.2
Annual income (Yuan)						
<10,000	262	31.4	111	28	149	48.1
10,000 to 40,000	388	46.5	231	58.3	151	48.7
≥40,000 to ≤80,000	43	5.2	35	8.8	8	2.6
>80,000	21	2.5	19	4.8	2	0.6

### 3.2. Lifestyle-related practices

#### 3.2.1. Smoking

Across respondents, 21% reported being frequent smokers (Table 2), 48% reported being non-smokers, and 32% reported smoking almost never/seldom/sometimes. The relationships between gender and smoking status was statistically significant (*p* < 0.001), with 27% of men and 74% of women reporting being non-smokers, 40% of men and 22% of women reporting smoking almost never/seldom/sometimes, and 33% of men and 5% of women reporting smoking usually/always. The same pattern of gender difference was apparent when asked about smoking as a means of managing stress (*p* < 0.001), with 27% of men and 62% of women reporting never doing so, 60% of men and 36% of women reporting almost never/seldom/sometimes; 13% of men and 2% of women reporting usually/always, respectively. Within smokers, men were more frequent users of pipes and cigars than women (*p* < 0.001).

**Table 2.** Smoking practices of respondents by gender (%).

		Gender		
		Men	Women	Total
Smoking status**	Never	26.5%	73.6%	47.6%
χ^2^ = 198.2; *p* < 0.001; df = 2	Almost never; seldom; sometimes	40.2%	21.7%	31.9%
	Usually; almost always; always	33.3%	4.6%	20.5%
How many cigarettes, cigars or pipe do you smoke in a typical day** (non-smokers are excluded)	Mean	8.11	3.04	7.01
*p* < 0.001 (Mann-Whitney U)	SD	9.11	5.85	8.76
On how many days of the week do you smoke**	Mean	3.24	.52	2.03
*p* < 0.001 (Mann-Whitney U)	SD	3.27	1.67	3.00
Do you live with or are you frequently around people who smoke**	Never	2.6%	6.0%	4.2%
χ^2^ = 16.7; *p* < 0.001; df = 2	Almost never; seldom; sometimes	59.0%	67.6%	62.8%
	Usually; almost always; always	38.3%	26.4%	33.0%

** *p* < 0.01.

No association between smoking status and education level was observed (*p* = 0.089). Within smokers, the difference of educational levels was significant in the number of cigars/pipes a respondent smoked daily (*p* = 0.003), with college/university diploma and high school being in one subset and primary school/no education being in another subset. Also, the number of cigars/pipes smoked per day by respondents with primary/no education was significantly higher than those consumed by respondents with education equal or greater than high school.

#### 3.2.2. Dietary practices

Table 3 shows respondents' daily consumption (g) of the basic food groups, and added salt and sugar. The basic food groups included whole grains, fruits and vegetables, meat and alternatives, and milk and dairy. In addition, when asked about their high fat fast food consumption, of 803 respondents, 51% (n = 428) consumed fast food less than once a week and 29% (n = 245) consumed it 1–2 times/wk.

With respect to BMIs, 74% of men and 67% of women were within the healthy range for the Asian population, i.e., 18.5 to 24.9 kg m^2^ (n = 831 reporting) [20]. 20% and 17% of males and females, respectively, were classified as overweight; few males (2%) and females (3%) met the WHO classification of obese. However, more females (13%) than males (4%) were classified as underweight.

**Table 3.** Dietary practices of respondents (descriptive statistics).

Food category		
Consumption	Mean (SD)	n (%)
Whole grains (g/day)	327.5 (231.65)	817 (97.8)
Fruit/vegetables (g/day)	282.1 (231.19)	811 (97.1)
Meat and alternatives (g/day)	180.7 (150.82)	805 (96,4)
Milk and dairy (g/day)	183.1 (152.05)	794 (95.1)
Oil products (g/day)	57.4 (66.99)	763 (91.2)
Sugar added (tsp/day)	3.9 (12.99)	765 (91.2)
Salt added (tsp/day)	3.7 (7.42)	772 (92.5)
High fat/fast food (x/wk)		803 (96.2)
<1x/wk		428 (51.3)
1–2x/wk		245 (29.3)
≥3x/wk		130 (15.6)

#### 3.2.3. Physical activity

Analysis of self-reported data on physical activity, exercise, and sitting over the 7 days immediately prior to data collection showed that the participant number within the sample sub-sets is variable given participants' inability to accurately recall the details of their physical activity, exercise, and sitting patterns. Total minutes of physical activity per week (moderate and vigorous) were calculated using the product of minutes per day and days/wk of physical activity engagement reported. In terms of physical activity for health benefits, 734 participants responded to the query of physical activity participation. Of these n = 456 (62.1%) met or exceeded the threshold for health benefits of 150 minutes of moderately-intense physical activity weekly and/or 75 minutes of vigorously intense physical activity (the n = 456 includes n = 134 who met both moderate and vigorous PA thresholds) [21],[22]. Median minutes for moderate physical activity and vigorous physical activity were 420 (n = 362) and 240 (n = 228), respectively.

Table 4 categorizes the respondents' physical activity, exercise, and sitting levels by gender over the previous 7 days.

Mann-Whitney tests showed that males spent more time than females performing vigorous exercise. For those who met physical activity thresholds, the Kruskal-Wallis tests showed no significant differences for weekly minutes of moderate or vigorous physical activity across levels of education (college/university, technical/high school or elementary/no education). However, in terms of time sitting, college/university educated spent significantly more time sitting compared to those with technical/high school education (*p* < 0.001) and those with elementary or no education (*p* < 0.001).

**Table 4.** Physical activity patterns of respondents over the past week by gender.

		Gender		
		Men	Women	Total
During the last 7 days, on how many days did you do vigorous physical activities? (n = 365)	Mean	2.9	2.9	2.9
	SD	2.0	2.0	2.0
How much time (hour/d) did you usually spend doing vigorous physical activities on one of those days (n = 365)*	Mean	1.4	1.1	1.3
	SD	1.4	1.1	1.4
During the last 7 days, on how many days did you do moderate physical activities? (n = 561)	Mean	4.0	4.2	4.1
	SD	2.2	2.3	2.2
How much time (hour/d) did you usually spend doing moderate physical activities on one of those days (n = 561)	Mean	1.4	1.5	1.5
	SD	1.4	1.5	1.4
During the last 7 days, on how many days did you walk for at least 10 minutes? (n = 724)	Mean	5.8	5.9	5.8
	SD	1.9	1.7	1.8
How much time (hour/d) did you spend walking on one of those days? (n = 724)	Mean	1.1	1.1	1.1
	SD	1.3	1.1	1.2
During the last 7 days, how much time (hour/d) did you spend sitting? (n = 761)	Mean	5.8	5.9	5.8
	SD	3.3	3.5	3.4

* U =13676.5, Z = −2.2, *p* < 0.05, r = 0.11.

Note: There is a discrepancy between those who met the physical activity requirements and the combined total of those who reported achieving thresholds for moderate and vigorous physical activity. Over 90 respondents reported engaging in both moderate and vigorous physical activity. We reported moderate and vigorous physical activity levels separately.

Table 5 shows the relationship of both walking and sitting variables with level of education, i.e., no formal education or elementary school; high school, or technical or trade school; college/university diploma or postgraduate degree. Higher education was associated with more days in the week and minutes/day of sitting (*p* < 0.001).

#### 3.2.4. Stress and sleep

Respondents were asked to rate common life stressors on a three-point scale, ranging from little to none (scored as 1) to a great deal (scored as 3) (Table 6). Of stressors, work- and health-related stress (and sickness in the family) affected over 60% of respondents and overall daily stress was reported to be moderately intense. Of the life stressors, personal and family sickness generally have larger impact on respondents, as about 55% of respondents reported a great deal of stress caused by sickness in family and 43% reported stress caused by personal sickness. No gender difference was detected of all the other stressors except sickness in the family (*p* = 0.045), women in general are more likely to be concerned about family members' sickness than men. Nearly 60% of respondents reported stress in their life was moderately intense, with 57% of men and 63% of women reporting overall stress to be moderate high.

Stress level and education were positively associated (*p* < 0.001), although the association is not strong. When the population is stratified into 4 age groups (18–34; 35–49; 50–64; ≥65 y), stress was observed to be negatively associated with age group (*p* < 0.001), with the youngest group (18–34 y) reporting most stress (Table 7).

**Table 5.** Walking and sitting patterns of respondents by education.

		Education			
		No formal education; elementary school	Technical or trade school; high school	College/university diploma; postgraduate degree	Total
During the last 7 days, on how many days did you walk for at least 10 minutes? (n = 725)	Mean	6.2	5.8	5.8	5.8
	SD	1.7	1.8	1.9	1.8
How much time (hours/d) did you spend walking on one of those days (n = 725)	Mean	1.3	1.1	1.1	1.1
	SD	1.5	1.2	1.2	1.2
During the last 7 days, how much time (min/d) did you spend sitting? (n = 725)*	Mean	4.4	4.9	6.6	5.8
	SD	3.1	3.4	3.2	3.4

* U = 7419.5, Z = −5.1, *p* < 0.001 between College/University/Post graduate and No Formal Education/Elementary.

* U = 40756.0, Z = −7.4, *p* < 0.001 between College/University and Technical/Trade/High School.

### 3.3. Lifestyle-related beliefs

With respect to respondents' beliefs about the importance of various lifestyle behaviors to overall health, Table 8 shows the results for men and women. Of all the items that were believed to be very/extremely important, not smoking cigarettes were ranked first by 64% of all respondents, followed by consuming plentiful fruit and vegetables by 61%, exercising regularly by 55%. Table 8 also shows the proportion of respondents rating each positive lifestyle-related behavior as not important. These were consistently less than 9% with one exception. Taking vitamins and mineral supplements regularly, with 22% of respondents reported it to be not important.

With respect to gender differences, women more than men believed that the following were more important: “eating a diet that is low in fat” (*p* = 0.021), “eating lots of fruit and vegetables” (*p* = 0.027), “not smoking cigarettes” (*p* = 0.002), and “maintaining a normal healthy body weight” (*p* = 0.007). Women are more likely to report diet and weight to be very important compared with men, and more likely to practice smoking abstinence than men. This latter finding was consistent with smoking practices in men and women.

**Table 6.** Stressors reported by respondents by gender (%).

		Gender		
		Men	Women	Total
Family/marriage (n = 789)	little to none	30.5%	31.1%	30.8%
	somewhat	33.0%	32.0%	32.6%
	a great deal	36.5%	36.9%	36.7%
Away from home (n = 757)	little to none	47.3%	47.6%	47.5%
	somewhat	41.3%	37.8%	39.7%
	a great deal	11.4%	14.6%	12.8%
Work (n = 792)	little to none	18.6%	25.5%	21.7%
	somewhat	43.3%	36.7%	40.4%
	a great deal	38.1%	37.8%	38.0%
Lack of work (n = 736)	little to none	30.7%	36.3%	33.2%
	somewhat	29.9%	30.5%	30.2%
	a great deal	39.4%	33.2%	36.6%
Few friends (n = 772)	little to none	33.9%	35.3%	34.5%
	somewhat	48.3%	49.0%	48.6%
	a great deal	17.8%	15.7%	16.9%
Sickness (n = 764)	little to none	18.8%	17.2%	18.1%
	somewhat	40.2%	37.0%	38.8%
	a great deal	40.9%	45.8%	43.1%
Sickness in family (n = 791)	little to none	12.6%	10.7%	11.7%
	somewhat	35.7%	30.3%	33.3%
	a great deal	51.6%	59.1%	55.0%
Family financial burden (n = 838)	little to none	100.0%	33.3%	50.0%
	somewhat		16.7%	12.5%
	a great deal		50.0%	37.5%

**Table 7.** Life stress level reported by respondents by age category (n, %).

	Stress level in your life							
	Low		Moderate		High		Total	
Age (y)	n	%	n	%	n	%	n	%
18–34	46	17.4%	190	71.7%	29	10.9%	265	100.0%
35–49	37	22.2%	114	68.3%	16	9.6%	167	100.0%
50–64	96	41.7%	115	50.0%	19	8.3%	230	100.0%
≥65	74	51.7%	62	43.4%	7	4.9%	143	100.0%
Total	253	31.4%	481	59.8%	71	8.8%	805	100.0%

**Table 8.** Respondents' beliefs about lifestyle-related behaviors (smoking, nutrition and exercise) and health status by gender.

		Gender		
		Men	Women	Total
Eating a diet that is low in fat*	Not important;	4.2%	2.7%	3.5%
	A little important; Somewhat important	53.9%	47.7%	51.1%
	Very important; Extremely important	41.9%	49.6%	45.4%
Eating lots of grains-based food	Not important;	7.8%	9.6%	8.6%
	A little important; Somewhat important	55.3%	53.7%	54.6%
	Very important; Extremely important	36.9%	36.6%	36.8%
Eating lots of fruit and vegetables*	Not important;	1.3%	.8%	1.1%
	A little important; Somewhat important	41.0%	34.0%	37.8%
	Very important; Extremely important	57.7%	65.2%	61.1%
Taking vitamins and herb/mineral supplements regularly	Not important;	20.7%	19.4%	20.1%
	A little important; Somewhat important	56.2%	60.7%	58.2%
	Very important; Extremely important	23.1%	19.9%	21.7%
Exercising regularly	Not important;	3.3%	2.2%	2.8%
	A little important; Somewhat important	40.0%	43.3%	41.5%
	Very important; Extremely important	56.7%	54.5%	55.7%
Not smoking cigarettes*	Not important;	5.8%	7.6%	6.6%
	A little important; Somewhat important	35.8%	22.0%	29.6%
	Very important; Extremely important	58.4%	70.4%	63.8%
Not drinking alcohol or drinking moderation	Not important;	6.3%	7.2%	6.7%
	A little important; Somewhat important	50.7%	43.8%	47.6%
	Very important; Extremely important	43.0%	49.0%	45.7%
Maintaining a normal healthy body weight**	Not important;	3.3%	2.0%	2.7%
	A little important; Somewhat important	47.2%	39.0%	43.5%
	Very important; Extremely important	49.5%	59.0%	53.8%

* *p* < 0.05, ** *p* < 0.01.

### 3.4. Concordance of lifestyle-related behaviors and beliefs, and with evidence-based recommendations for maximal health

We examined three primary relationships between lifestyle-related practices/behaviors and beliefs about these and maximal health, specifically, relationships between smoking practices and belief about smoking and health; between calculated BMI and belief about healthy BMI for health; and level of physical activity and belief about the importance of physical activity/exercise for health. We observed a positive association between smoking practice (categorized as never, almost never/seldom/sometimes, usually/almost always/always) and the belief that not smoking cigarettes is importance to health (Kendall's tau-b = −0.211, *p* < 0.001; n = 824). Specifically, those respondents who believe that ‘never smoking cigarettes’ is important to health, generally don't smoke or smoke less than those who believe that not smoking cigarettes is of low importance to a person's overall health.

No association was observed between a respondent's calculated BMI and that individual's belief about the importance of maintaining a healthy body weight (Kendall's tau-b = 0.018, *p* = 0.609; n = 791). However, we did observe a negative association between a respondent's weekly frequency of fast food consumption vs. that individual's belief about the importance of consuming a low fat diet for health (Kendall's tau-b = −0.063, *p* = 0.039; n = 803).

Although alcohol consumption was generally low in this Chinese cohort, when respondents were categorized in terms of drinking risk (based on Canadian standards of more than one glass of wine daily for women, for example, and two for men), there was no association between level of drinking risk and respondents' beliefs about the importance of drinking in moderation or not drinking, for health benefit (Kendal's tau-b = −0.016, *p* = 0.664; n = 768).

Regarding, physical activity, no association was observed between those who participated in the recommended guidelines for physical activity (categorized as Yes/No) and belief about the relative importance of physical activity/exercise is for general health (Kendall's tau-b = 0.019, *p* = 0.624; n = 723). Alternatively, the belief that exercising regularly is important to health is not necessarily associated with whether respondents met the physical activity or exercise guidelines for good health.

## 4. Discussion

Given the tenets of the Health Belief Model [9] regarding the need to focus on lifestyle-related health beliefs as well as practices when developing, targeting, and implementing health education programs, we examined the concordance of practices with beliefs, and their concordance with evidence-informed guidelines of maximal health in Chinese urban dwellers. Our findings support discordance between their lifestyle-related behaviors and beliefs, and between these behaviors and beliefs and evidence-informed guidelines for health, i.e., the singular importance of not smoking; consuming a well-balanced diet daily consisting of several servings of multi-grains, and fruit and vegetables, and limited consumption of meat, fats and oils, and sugar [18],[19]. We discuss the findings under the lifestyle-related categories, namely, smoking, dietary practices, physical activity, and stress and sleep.

### 4.1. Smoking

Smoking practices and beliefs about smoking being culturally acceptable and desirable persist with China remaining a country with a high proportion of smokers [17]. No amount of smoking is recommended or acceptable for maximal health [16]. Chinese men remain heavy smokers [17]. Women in our sample are generally non-smokers and more likely to view non-smoking as very important to health. However, China is characterized as a masculine culture [25], which is consistent with men's positive attitude toward smoking in contrast to women's, hence its prevailing acceptance within the family and society overall. Consequently, Chinese non-smoking women are exposed to passive smoking and its risks.

### 4.2. Dietary practices

Until recent decades, China was held in regard with respect to healthy living indicators including healthy unadulterated nutrition (the Asian advantage) [26]. The health of the people of China however has been changing rapidly commensurate with its economic development [1]. The traditional Western diet, known not to be healthful and associated with NCDs, has become increasingly prevalent in China, particularly in urban areas [26]. The findings from the present study support deficiencies in the lifestyle-related health behaviors of mainland Chinese, and the gap between healthy lifestyle-related behaviors and beliefs of the Chinese sample surveyed. In addition, we observed gaps between established evidence-based recommendations for health living, and the practices and beliefs of our sample.

Based on nutritional guidelines for the Chinese [18] the daily eating practices of our respondents were generally consistent with those that are recommended for general health with some exceptions. Added sugar and salt were high, and almost half were eating high fat/fast food regularly (375 or 44.9%), i.e., at least once a week.

The healthy BMI range for the Asian population is 18.5 to 24.9 kg m^2^ [20]. Of the urban Chinese we sampled, a proportion of both men and women above and below this range suggesting the need for preventive health programs targeted at the individual as well as the population.

Chinese healing traditions have included herbs and supplements for centuries [27]. Although 58% of respondents believed taking these was a little to somewhat important to overall health status, 22% believed they were very or extremely important. Although there are reasons for which supplements or herbs are indicated for an individual, the question was related to their use for maintaining good health ‘in general’. Generally speaking therefore, one could argue that supplements are not needed if an individual has a healthy well balanced diet.

The traditional role of women being responsible for the diet of the family may partly explain gender differences related to nutrition [27]. In turn, because nutrition is fundamental to health and poor nutrition is a well-established independent risk factor for lifestyle-related NCDs, this difference might further explain women's superior health indicators.

### 4.3. Physical activity

For optimal health, 150 minutes of moderately-intense physical activity weekly or 75 minutes of moderately intense activity are recommended [21],[22]. Prolonged uninterrupted periods of sitting daily are to be avoided [22]. People with lower education can be expected to be employed in more physically demanding work and labor, compared to those with higher education [28]. This was supported by our findings. Furthermore, those with higher education reported sitting more which is consistent with those educated participants holding white collar jobs. Interestingly, in western countries, worker and occupational safety has focused on jobs requiring manual labor and in construction and mining. This perspective in contemporary times needs to be reconsidered given the documented hazards of work-related prolonged sitting. Although our data showed 62.1% of respondents are engaged in physical activity that met the threshold for health benefits, over one third (37%) of the total sample are engaged in jobs that require sitting for over 5 hours a day. It is possible that even though mean BMI is within an acceptable range, current work patterns may contribute to an upward trend of increased daily sitting consistent with trends in high income countries.

### 4.4. Stress and sleep

Western living coupled with traditional values in China is associated with moderate to high levels of stress [29] and increasingly compromised sleep, itself a correlate of NCDs [23]. The injurious nature of unrelenting stress and how its negative effect on sleep is often underappreciated [23] with individuals often believing that sleep is a dispensable luxury. Our findings help to identify the prevalence of stress is a large sample of urban Chinese, and how beliefs about stress and its reduction, and about sleep and its optimization for health can be addressed. Cultural factor may be at play given the Confucian work ethic, hence negative attitudes toward recreation and play [30].

### 4.5. Relationships among lifestyle practices and related beliefs for maximal health

Health professionals may assume that if a person has accurate beliefs about the relationships of lifestyle practices such as smoking, healthy diet and weight, and healthy physical activity levels, that these would lead to healthy lifestyle practices. Our preliminary findings showed inconsistent relationships. They suggest that beliefs about the health consequences of various lifestyle practices can be distinct to what a person actually practices. Further study is needed to examine these relationships in greater detail in the cohort of interest in this study and in the general Chinese population. If valuable resources are to be invested into health promotion education campaigns, it is essential that programs are tailored and targeted to the needs of individuals and groups. For example, people's health and their knowledge about NCDs, their lifestyle practices which can be readily assessed clinically with tools such as the Health Improvement Card advocated by the World Health Professions Alliance (www.whpa.org), and their beliefs about lifestyle and health and NCDs, provides essential information in identifying what type of health education needs to be delivered and at what level, i.e., knowledge, practice or belief, or some combination.

Finally, in addition to lifestyle beliefs and practice, attention needs to be given to the issue of motivation and adherence to recommendations to be more active and participate in regular physical activity. Rhodes and Fiala have addressed this issue. They have provided recommendations to maximize motivation. Such recommendations need to be evaluated in the Chinese context [31].

### 4.6. Strengths and limitations

This is the first study aimed at the mainland Chinese population and burgeoning NCD prevalence, with a view to identify the gap between knowledge, and lifestyle beliefs and practices, and the potential to reduce it by targeting health education accordingly. This being a first-of-its-kind study is a strength, given it provides a starting position for other studies including sampling frame considerations and sample size calculations. Specifically, our findings have implications for the design, development and implementation of health protection and promotion campaigns in China. In addition, the knowledge gleaned in this study can also help inform the requisite competencies needed by contemporary health professionals in their practices [32]. Second, questionnaire studies are relative easy to administer with trained personnel, and can be far reaching.

With respect to limitations of the study, although the sample size in our study was over 800, snowballing sampling rather than random sampling may have resulted in the age, education, and income distribution that may not be typical of the general public in China. Our sample reflected the characteristics of generally educated Chinese, given that most Chinese are classified as less educated and in the low-to-middle income bracket [1]. Further, in the urban areas of the four provinces in which participants were sampled in this study may not be representative of the Chinese population in other urban areas, or the rural areas. Further research is needed to replicate and extend these findings to clarify these points. Finally, we used standardized questions to capture physical activity and activity behavior of participants. Because they could respond to more than one category of physical activity/exercise, i.e., moderately intense and moderately vigorously intense, variations in sample sizes resulted across levels of physical activity and exercise. Further, the percentage of participants who met physical activity guidelines (62.1%) may be inflated given n = 101 missing values for those who did not respond to PA questions.

## 5. Conclusion

Given the progressive escalation of lifestyle-related NCDs in China, evidence-informed targeted health education programs for individuals and the public are warranted. Our study provides new evidence about the lifestyle-related health behaviors and beliefs of a large sample of urban mainland Chinese. Our findings highlight discordance not only between their practice and beliefs, but also between these and evidence-based recommendations for health. We conclude that for mainland urban Chinese to meet the established recommendations for healthy living (i.e., not smoking, consuming a healthy diet and maintaining a healthy weight, being regularly physically active with reduced prolonged periods of sitting, and experiencing manageable stress and quality sleep), lifestyle-related knowledge and beliefs as well as practices need to be foci of health education and population-based health promotion campaigns if these are to be cost-effective. Future studies are needed to replicate and extend these observations to other cohorts of the mainland Chinese population including issues related to motivation, adherence and commitment to being physically active.
